# Can a chatbot enhance hazard awareness in the construction industry?

**DOI:** 10.3389/fpubh.2022.993700

**Published:** 2022-11-30

**Authors:** Xiaoe Zhu, Rita Yi Man Li, M. James C. Crabbe, Khunanan Sukpascharoen

**Affiliations:** ^1^Chakrabongse Bhuvanarth International Institute for Interdisciplinary Studies, Rajamangala University of Technology Tawan-Ok, Bangkok, Thailand; ^2^Sustainable Real Estate Research Center, Hong Kong Shue Yan University, Hong Kong, Hong Kong SAR, China; ^3^Wolfson College, Oxford University, Oxford, United Kingdom; ^4^Institute of Biomedical and Environmental Science and Technology, School of Life Sciences, University of Bedfordshire, Luton, United Kingdom; ^5^School of Life Sciences, Shanxi University, Taiyuan, China

**Keywords:** eye-tracking, construction hazard awareness, construction practitioners, design of experiment, chatbot safety training

## Abstract

Safety training enhances hazard awareness in the construction industry. Its effectiveness is a component of occupational safety and health. While face-to-face safety training has dominated in the past, the frequent lockdowns during COVID-19 have led us to rethink new solutions. A chatbot is messaging software that allows people to interact, obtain answers, and handle sales and inquiries through a computer algorithm. While chatbots have been used for language education, no study has investigated their usefulness for hazard awareness enhancement after chatbot training. In this regard, we developed four Telegram chatbots for construction safety training and designed the experiment as the treatment factor. Previous researchers utilized eye-tracking in the laboratory for construction safety research; most have adopted it for qualitative analyses such as heat maps or gaze plots to study visual paths or search strategies *via* eye-trackers, which only studied the impact of one factor. Our research has utilized an artificial intelligence-based eye-tracking tool. As hazard awareness can be affected by several factors, we filled this research void using 2-way interaction terms using the design of experiment (DOE) model. We designed an eye-tracking experiment to study the impact of site experience, Telegram chatbot safety training, and task complexity on hazard awareness, which is the first of its kind. The results showed that Telegram chatbot training enhanced the hazard awareness of participants with less onsite experience and in less complex scenarios. Low-cost chatbot safety training could improve site workers' danger awareness, but the design needs to be adjusted according to participants' experience. Our results offer insights to construction safety managers in safety knowledge sharing and safety training.

## Introduction

Construction safety is a long-term global problem (1, 2), with 75% of European non-fatal work injuries happening in the construction industry (3). Human errors account for 80% of construction incidents (4). Cognitive psychologists believe human error results from one or multiple failures in human cognition in hazard perception, recognition, and decision-making (5). While hazard recognition largely depends on workers' ability to detect hazards (6), individuals' working experiences enhance hazard awareness (7). Safety training reduces the possibility of accidents in the construction industry (8, 9), and its effectiveness is a component of occupational safety and health (10). Previous research found that an American 10-hour Occupational Safety and Health Administration hazard awareness training program improved the workers' attitudes toward safety (11, 12). While face-to-face or onsite safety training was typical before COVID, confirmation of human-to-human COVID-19 transmission *via* droplets and contact (13) has led to many lockdowns and an increase in the need for online and mobile training.

Even when most countries have returned to near normal, online mobile training that allows us to receive knowledge at any time and place has already become the norm. Compared to traditional face-to-face training, it can overcome a problem when many trainees are needed simultaneously. Thus, some construction safety training is designed to allow individuals to learn through mobile phones. For example, the US utilizes Wireless Information System for Emergency Responders (WISER) to share construction knowledge related to potential explosive activities and events on sites (14). Various Web 2.0 tools can be accessed *via* mobile phones like social networks Wikipedia (15), YouTube (16), Twitter (17), and Weibo (18). In 2022, Telegram was in the top-5 list of downloaded apps globally, and it today boasts more than 700 million active monthly users (19). While making a Telegram AI bot is free, and users can use it for free anytime and anywhere, no research has studied the use of Telegram for construction safety training to the best of our knowledge. This research investigates the possibility of using Telegram to increase hazard awareness.

Eye tracking is an excellent tool for measuring situational awareness (20). Researchers introduced this technology to study construction safety and evaluate the impacts of workers' hazard-identification skills on their attention distributions and visual search strategies *via* a qualitative approach or descriptive statistics by categories (21, 22), which do not provide a sufficient exploration or explanation of the data. Most researchers have designed experiments with lab eye-tracking equipment with only two or three data types, but not online eye-tracking with AI for more data types (23). Partly due to the co-existing requirements of programming, eye tracking, and modeling knowledge, no research has been used to test the impact of Telegram chatbot safety training on construction practitioners' hazard awareness *via* eye-tracking or analyzing eye-tracking data with a DOE orthogonal design approach. Our research attempts to fill this gap.

In most of the previous research, the experiment participants were students (24–27). Nevertheless, students' results may not completely or accurately reflect the behaviors of construction practitioners. In this paper, our experimental participants come from the construction industry, so the results are close to reality. We aimed to study Telegram chatbot safety training to enhance construction practitioners' hazard awareness measured by AI-based eye-tracking. Most eye-tracking experiments have used qualitative and descriptive analysis (23). Our full factorial design of the experiment processes data with three factors, allowing researchers to analyze the factors' main and interactive effects.

Our novelty is as follows: (1) we experimented with AI-based online eye-tracking for testing construction hazard awareness; (2) we investigated the enhancement of construction practitioners' hazard awareness after Telegram chatbot safety training; and (3) we processed the safety awareness data based on the full factorial design of the experiment (DOE) to see the impacts of multiple factors on the effectiveness of Telegram chatbot safety training. The remainder of this paper is structured as follows: Section 2 is the Literature review that discusses the theoretical background and current research; Section 3 lays out the experimental design details and method; Section 4 presents the results and the data analyses; Section 5 is the discussion; Section 6 summarizes the study, notes the limitations, and suggests the scope for future research.

## Literature review

### Construction hazard awareness

Utilizing situation awareness (SA) theory, Endsley (28) explored the relationship between participants and the environment. It suggested that when a person encounters a dangerous situation, correct and quick decision-making involves pattern recognition or matching, which requires the formation of sophisticated schemata, and prototypical conditions that facilitate the decision-making process (29). One human factor that leads to the frequent exposure of construction workers to hazardous environments is their lack of situational awareness. Situation awareness remains an abstract concept due to the difficulty in measuring and quantifying the situation awareness of any worker (30). To improve safety on construction sites, workers must be aware of activities and elements within the work environment (31). The situation awareness for hazard recognition for construction workers or engineers is also known as hazard awareness. Eye-tracking is a technology that can obtain participants' observed viewing patterns, understand what they are interested in (27), and gather their conscious and unconscious data within a few minutes. Hasanzadeh, Gad (22) and Kaber, Riley (32) showed that eye-tracking is a subjective safety awareness measurement for the hazard awareness of construction workers.

### Chatbots' safety training, experience, and complexity of the task

Hasanzadeh, Esmaeili (33) concretize Endsley (34)'s model into individual factors (abilities and knowledge, experience, training, goals, and expectations) and external factors (workload, stress, automation complexity of the task) as aspects for study in the construction hazard condition. Work experience is highly correlated with hazard awareness (35). It could assist construction workers or organizations in improving their safety performance (36, 37) and affect their ability to identify hazards (38). Nevertheless, it is unavoidable that workers start with zero construction work experience on-site. Thus, how can we raise new workers' hazard awareness? Likewise, continuing professional development by attending safety training among experienced workers is essential to maintain their safety awareness. Are there any means that can fulfill this?

Safety training manuals improve workers' hazard awareness by improving their safety knowledge and skills to reduce the probability of construction accidents (8, 9). Hundreds of research articles have focused on evaluating or developing effective safety interventions (11, 39). These include safety training to enhance hazard recognition (40). With the development of digital technology, cell phone ownership and smartphone-enabled technologies have become popular among construction workers worldwide (41), and chatbots have been used to handle inquiries. It does not require professional technical support, and users only need a smartphone. It can be used anytime and anywhere and is low-cost and straightforward (42). Chatbots, software avatars with a limited but increasing capability to chat with humans, are good learning enhancement tools outside the construction industry (43, 44). Burke and Sarpy (45) suggested that safety training involving human dialogue is more effective than traditional medicine and psychology lectures. The application of chatbots in various industries needs empirical research (42). Our previous studies proposed using simple Vbot chatbot applications to share construction safety knowledge (46).

As workers' experience, age, and physical conditions are always highly correlated in construction safety studies (47), prior research that combines these factors in the same study is limited (48), not to mention hazard awareness after Telegram chatbot training. This research aims to study various factors affecting construction hazard awareness using eye tracking.

### Experiment participants and eye-tracking data processing method

Pernice and Nielsen (49) suggested that 6 people for a qualitative research study and 30 for quantitative experimental research would be sufficient in an eye-tracking study. Xu and Chong (24) recruited 47 students in different grades (34 male, 13 female); de la Fuente Suárez (27) recruited 40 students. In reference to these studies, we recruited volunteers from the construction company of onsite workers, design engineers, and novice engineers on site.

Pernice and Nielsen (49) suggested analyzing gaze plots, heat maps, areas of interest, video, and animation for eye-tracking experiments. For the eye-tracking experiment studying construction hazard awareness, de la Fuente Suárez (27) utilized the heat-maps analysis approach for studying real-world visual attention to a historic building. Han, Yin (26) used the eye metrics value to compare the descriptive statistics alongside the maps. Dzeng, Lin (7) adopted eye-tracking metric fixation numbers and compared inferential statistics. Hasanzadeh, Esmaeili (35) used maps and eye-tracking metric fixation count and fixation duration to discriminate between different hazard types. Furthermore, Hasanzadeh, Esmaeili (50) analyzed the working-memory effects on hazard awareness or recognition ability. These studies usually only focused on a single factor's influence on the eye-tracking result. There was no other experimental design and analysis to combine multiple factors. Most of the data processing methods focused on the maps using qualitative analysis, eye-tracking data descriptive statistics, or a one-way variance test.

Our research adopts the DOE full factorial design method for data processing. A full factorial experiment is an experimental design that consists of two or more factors, each with discrete possible values or levels, and experimental units take all possible combinations of these levels across all aspects (51). Such a full factorial experiment allows the investigator to study the effect of each factor on the response variable and the impact of interactions between factors on the response variable (52).

### Ebbinghaus' forgetting curve and SF-12V2 scale

Ebbinghaus' forgetting curve is the famous for studying the human rate of forgetting. As time passes information loss can be very rapid and then levels off. After 2–6 days, retention of residual memory is only 28–25% (53), though this figure depends on the learned materials (54). We assume similar results at the end of 5 days of the Telegram chatbot safety training if the training cannot raise the residual memory. If the research participants accepted the chatbot construction safety training and their memory retains more than 28–25%, as revealed in eye track eye-tracking tests, this approach would be helpful for training.

The participants' situational awareness relates to their mental and physical states (34). To study other factors' effects on hazards, it is necessary to be clear about the physical and mental states of the participants (55). The SF-12V2 is among the most widely used classical scale for general health status measures. SF-12V2 can provide estimates for all eight domains and focuses more on Physical Component Summary (PCS) and Mental Component Summary (MCS) (56). Our study conducted a self-filled questionnaire analysis for participants' mental states, controlling for the effective participants with mental states with no significant differences.

## Research method

### Experimental factor: Chatbot safety training

The construction industry has reached some consensus (57): the most common property maintenance injuries on construction sites, ranked by the percentage of accidents, are: falling from a height, object strike, and electric shock (58–60). In particular, falling from a height is the first occupational injury in construction worldwide, and falling from a height is related to various forms of construction site border protection (59–62). We created four chatbots in the Telegram app for smartphones *via* @BotFather and python-telegram-bot, involving municipal construction, housing construction, civil engineering, and property maintenance (@Hksyubot, @refurbishmentbot, @newbulidingbot, and @constructionanswerbot). One can find it on the Telegram chat application in both English and Chinese, and each chatbot has four images of site hazards. All chatbots contain one hazard photo about falling from height @Hksyubot shows a photo with many vehicles besides road repairing, some workers do not wear reflective color clothes. The second photo with the old and exposed electrical wire may lead to electric shock. The third photo shows a worker who works at height without hard hat and safety belt. The last one is a photo with someone who cuts wood but do not wear the safety glove. @Refurbishment bot contains a photo with a site without safety net, a worker who does not wear hard hat, a photo that shows a worker who do not wear protective shoes. @Constructionanswerbot consists of a photo with too many construction workers work on a bridge, Electric wire on the tunnel floor which may lead to trip and fall and incorrect usage of a container as the support. Participants talk to the chatbot by themselves, which takes approximately 5–10 min.

**Figure 1:**
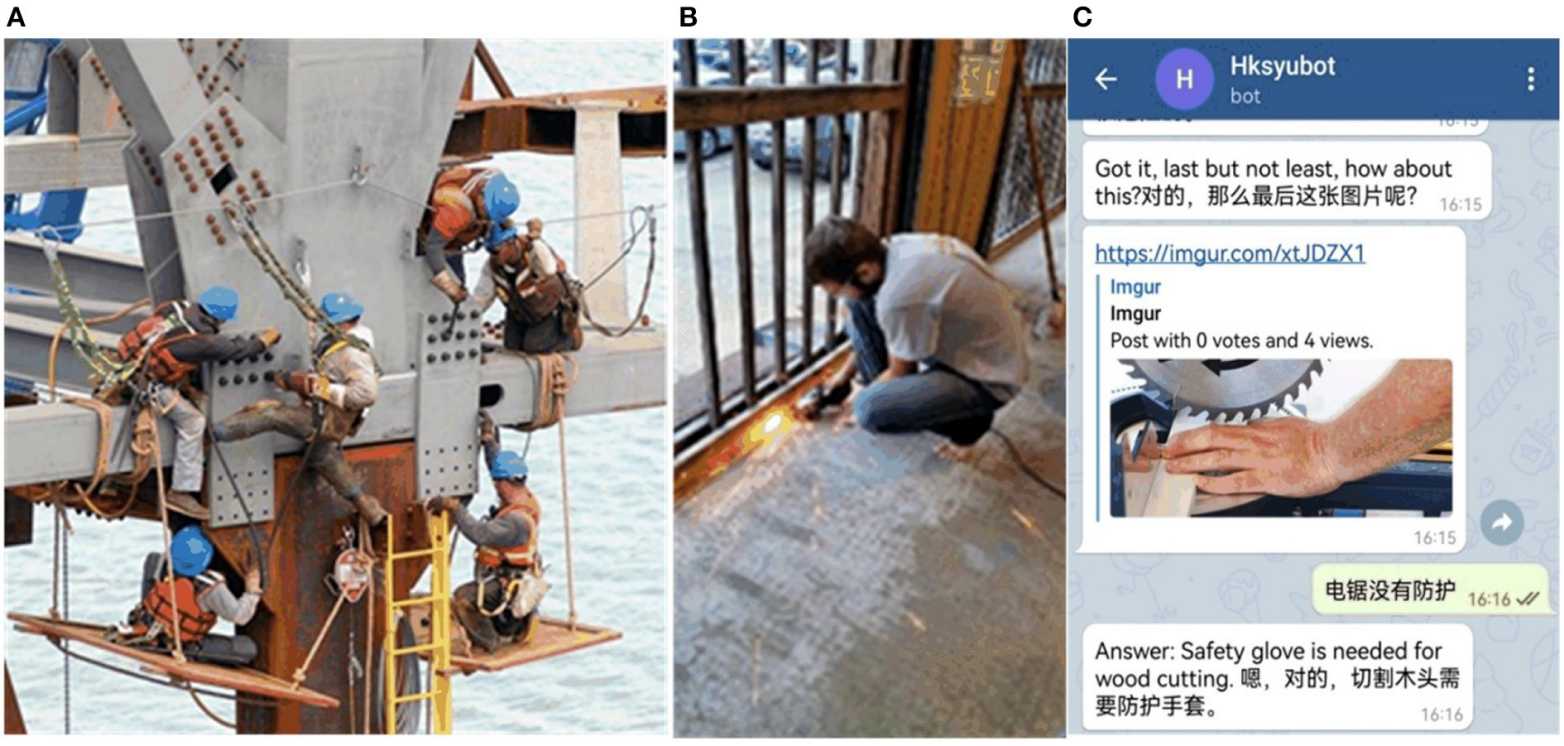
**(A–C)** Telegram chatbot safety training picture.

Figure 1 shows two representative training images and a screenshot of the chatbot. The chatbots asked questions about the hazards in each photo, as depicted in Figures 1A–C (complete sets of questions can be accessed by joining the abovementioned telegram bots), and the participants answered the questions. Then the bots inform the participant of the danger in the picture, such as in Figure 1A the offshore operation platform is unsafe, Figure 1B the operation of the grinder while barefoot and without protection, and Figure 1C the lack of chainsaw protection. In our experiment, the half of the participants that received chatbot safety training were the experiment group, and the other belonged to the control group.

### Experimental procedure and AI eye-tracking test

#### Experimental procedure

Our experimental process included three steps: First, researchers interviewed on the construction site, recruited volunteers, and talked with and informed the volunteers of the information on experimental content and research ethics. Second, the volunteers filled in the background information and SF-12V2 questionnaire, and half of the volunteers underwent chatbot safety training. Third, on the fifth day, all participants took an eye movement test. Finally, we operated the experimental platform to collect eye-tracking maps and data.

#### AI eye-tracking test

We conducted the experiments by using a remote AI eye-tracker. Compared with traditional laboratory equipment, the AI eye-tracker is relatively simple and convenient, and the experimenters do not need to receive special professional training. Without any other device, it only requires a computer to load the Cooltool platform. Participants sit comfortably in front of a computer, close to the bright windows, to ensure enough light. The experiment takes approximately 3 min with a good computer network. Participants can not wear glasses, walk, or cover their faces, and do not talk with each other in the process. The eye tracker collected data with a sampling rate of 30 Hz, meaning we received 30 data points per second.

**Figure 2:**
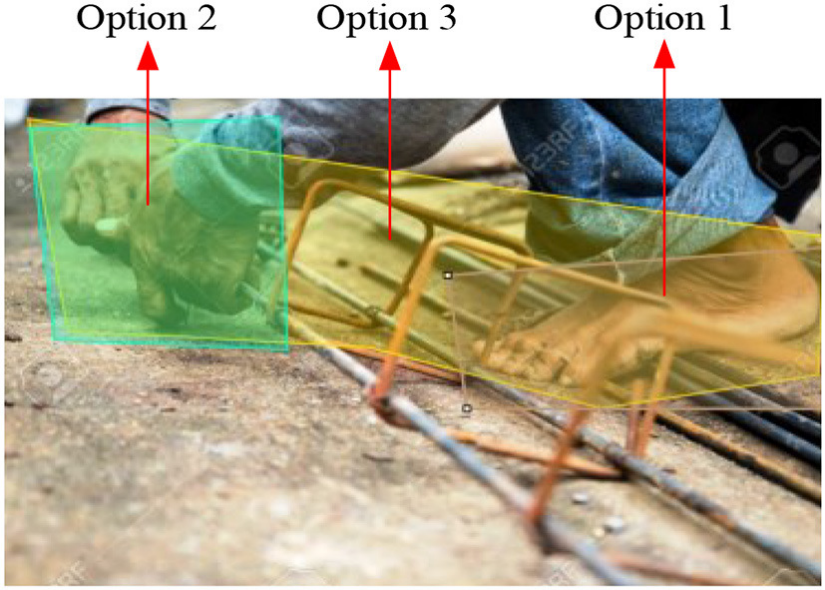
Test A and AOI options.

**Figure 3:**
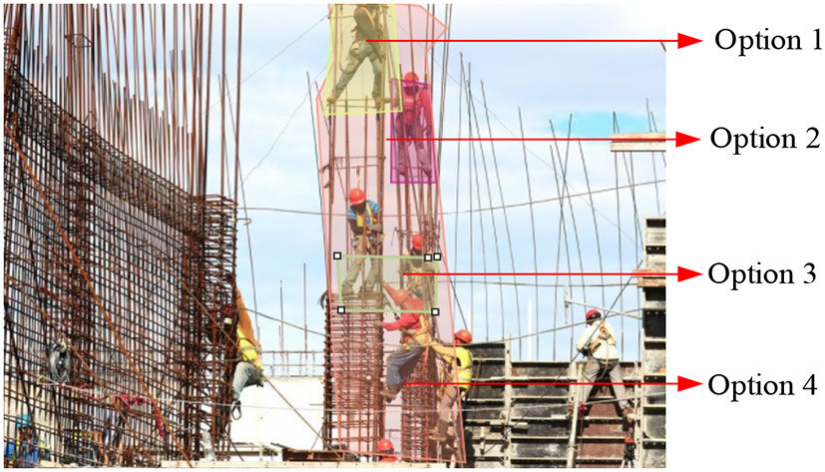
Test B and AOI options.

Volunteers took tests A and B to identify and determine the hazards shown in the photo with 10 seconds per picture. In test A, the worker did not wear gloves and shoes, which was a hazardous operation (Figure 2). Test B was more complex as there were many more activities and workers stepping on steel reinforcement in violation of the construction safety regulations (Figure 3).

#### Area of interest and eye-tracking metrics

The first step was to define the Area of Interest (AOI) in each test image, which was drawn as Options in The Cool tool platform to obtain the eye tracking maps and data from the online system. AOI indicates the hazards that the research participants identify. In each test, we selected some small and accurate local-scale AOI to obtain qualitative information and a larger-scale AOI for getting eye-tracking data for quantitative analysis. In Test A, Options 1 and 2 were local-scale AOI, and Option 3 was the wide-range AOI (Figure 2). In Test B, Options 1, 2, and 3 were local-scale AOI, and Option 4 was the wide-range AOI (Figure 3). Second, the background algorithm of the AI eye-tracking platform automatically calculated the metrics for this AOI, including fixation count, fixation duration time, time to the first fixation, and so on. The fixation count demonstrated how many respondents fixated their gaze on the selected object at least once. Fixation duration time reported how long a respondent looked at AOI in seconds. It recorded when a person fixated their gaze on the AOI and outside the AOI. Time to the first fixation is a metric that reflects the time it took a respondent to fix their sight on the selected AOI. The validity of the data was automatically determined by the eye-tracking platform system (https://app.cooltool.com/srvz8jztem?nl=true), test A, and test B.

In addition, this paper compared the test values under different factors with different conditions (levels). As some eye-tracking metrics were relatively small, the differences among all participants were small, and the absolute value of the test was not our main concern.

### Participants

#### Group information of participants

We designed our experiment with three groups of volunteers.

The participants in Group 1 included 10 valid test results from engineers from a project design company. Seven worked in architectural or structural design, two were in the project budget, and one was the project data manager in a design enterprise. They were aged between 40 and 50 years old. Except for one, who had worked for 1–2 years, all had worked for more than 10 years. Most of the time, they worked indoors, but sometimes they were on a construction site and familiar with construction site work.

Group 2 included 17 novice engineers onsite and 14 results were valid. They were young construction engineers and supervisors of engineers in their 20s. They had just graduated from university and completed civil engineering-related courses but lacked practical work experience.

Group 3 included 24 on-site workers; 14 results were valid. They were the tower crane drivers (the personnel who operate the tower crane machine at a high altitude on the construction site), the tower crane commander (the personnel who cooperate with the tower crane driver, work on the ground on-site, and send instructions to the tower crane driver), two safety managers, and one project manager. They all worked outdoors on construction sites with rich onsite experience. Most of them only had a middle school education but had professional operation qualification certificates.

#### Participants' general health and mental conditions

This study showed no significant differences in psychological and mental test scores, as in the study of Lam, Lam (63). We conducted the analysis using the Tukey Method and 95% Confidence with Minitab software for the SF-12V2 scores in Tables 1, 2; grouping means that do not share a letter are different. The Physical Component Summary of Groups 1 and 2 differed significantly because of age. The Mental Component Summary of all participants in this experiment was not significantly different.

**Table 1:** PCS: Grouping information.

**Group**	***N***	**Mean**	**Grouping**	
2	14	55.71	A	
3	14	52.56	A	B
1	10	49.12		B

**Table 2:** MCS: Grouping information.

**Group**	***N***	**Mean**	**Grouping**
1	10	48.38	A
2	14	47.55	A
3	14	46.3	A

### Data analysis model

Fully crossed factorial designs explore all possible levels of a given set of factors, and they can provide a condensed summary of the factor effects, simplifying the interpretation of factorial designs. It has been applied in agricultural research (64) and legal psychology (65). The orthogonal factorial analysis model is *Y* = μ+*AF*+ε, where $Y={\left(Y_{1},\ldots Y_{P}\right)}^{{}^{\prime }}$ are observable variables, and *F*_1_, …*F*_*m*_ are common factors of Y. The orthogonal factorial analysis model uses the linear combination of a few common factors to describe the change of variable *Y* (66) and extracts the variance of common elements. The eigenvalue is shown in a scree plot to indicate the number of significant factors (67, 68).

The design of the experiment (DOE) full factorial experimental model is *y* = *f*(*x*_1_, *x*_2_…, *x*_*k*_)+ε. Where *y* is a response variable, *x*_1_, *x*_2_…, *x*_*k*_ are controlled factors, *f* shows a specific functional relationship, and ε is an experimental error (69). A full factorial experiment shows that all factors combine at all levels for testing. All the main effects and interaction effects can be estimated. It is suitable for a small number of factors for testing (70). The DOE linear model includes not only one-time terms but also 2-way interactive terms *x*_1_*x*_2_, *x*_2_*x*_3_, *x*_1_*x*_3_. The response (dependent) variables always need to do box-cox trans formation and auto optimal value λ. In this experiment, in Minitab, the response variable y is transformed to fit the linear model (67), obtaining the y^*^ = (*y*^λ^−1)/(λ*g*^λ−1^) (λ = 4, *g* = 5.63961 is the geometric mean of *y*) with y^*^ as a new response variable for regression analysis.

According to the central limit theorem, if the test samples conform to a normal distribution, the minimum sample number of the experiment is greater than or equal to 5. Then, the mean value can meet the requirements (71). In the follow-up data processing of this experiment, the minimum group sample size was 5, and the average value of 5 or 7 participants was taken as a statistic for DOE full factorial experiment analysis. We utilized Stata 16 and Minitab 19 statistics software for analysis.

## Results

### Heat maps

**Figure 4:**
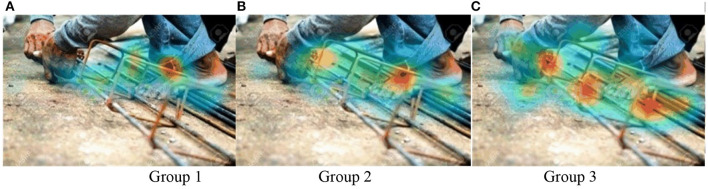
**(A–C)** Heat maps of all groups in Test A.

**Figure 5:**
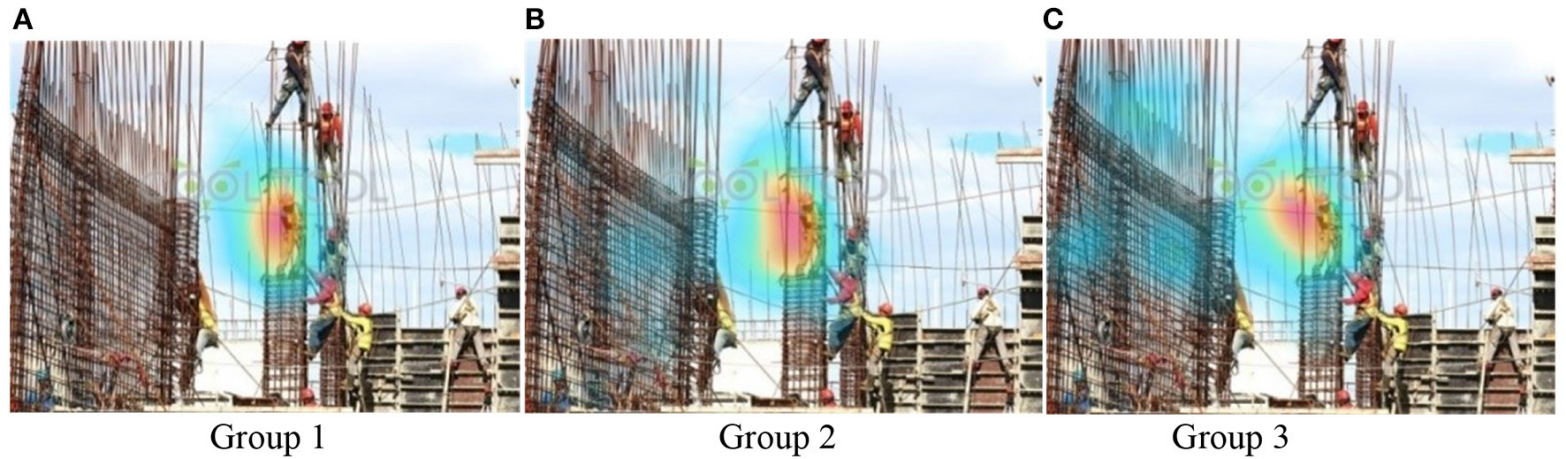
**(A–C)** Heat maps of all groups in Test B.

Heat maps (Figures 4, 5) show that Group 3 (Figures 4C, 5C), with rich site experience, has a broader focus. Group 3 on-site workers tended to be distracted when they viewed pictures, looked for other dangers besides the dangerous primary operations at the scene, and showed more confidence in identifying hazards. For some detail, in Test B, Option 1 (in Figure 3) is at the highest point. The participants' fixation count in Group 1 was 29.4%. Group 3's fixation count was 33.30%, significantly higher than Group 2, with 12.5%. This means the Group 2 novice engineers have a narrow and cautious focus. The general conclusion obtained by heat maps will be verified by quantitative analysis.

### Response variable (dependent variable) analysis

#### Response variable

Eye-tracking data from 76 participants were obtained from AOI Option 3 in Figure 2 and Option 4 in Figure 3. We analyzed the relationships of these data to choose the fitness metric for the response variable to undertake the design of experience (DOE) factorial analysis. We used STATA to analyze the correlation. The results are shown in Table 3. Most of the eye-tracking data were highly correlated with *p* < 0.01, so we selected one for DOE experimental analysis. Fixation count and observation count are counting numbers. Fixation duration time is correlated with high accuracy and with other time metrics (time to the first fixation, the time before the first fixation, and observation duration), the correlation coefficient with observation duration was a high value, 0.987. As Dzeng, Lin (7) used fixation number, and Hasanzadeh, Esmaeili (35) used fixation count, we chose fixation duration time (time length in seconds) as the response (dependent) variable for DOE factorial analysis.

**Table 3:** Pairwise correlations of eye-tracking metrics.

**Variables**	**(A)**	**(B)**	**(C)**	**(D)**	**(E)**	**(F)**	**(G)**	**(H)**	**(J)**
A	1.000								
B	0.845^***^	1.000							
C	−0.725^***^	−0.761^***^	1.000						
D	−0.738^***^	−0.783^***^	0.987^***^	1.000					
E	0.834^***^	0.965^***^	−0.763^***^	−0.791^***^	1.000				
F	0.080	−0.110	−0.253^**^	−0.225^*^	−0.183	1.000			
G	−0.074	−0.028	0.025	0.032	−0.046	−0.038	1.000		
H	0.309^***^	0.371^***^	−0.442^***^	−0.454^***^	0.367^***^	0.080	0.790^***^	1.000	
J	0.466^***^	0.431^***^	−0.541^***^	−0.541^***^	0.454^***^	0.155	0.316^***^	0.580^***^	1.000

^***^p < 0.01,

^**^p < 0.05,

^*^p < 0.1.

*A*, Fixation count; *B*, Fixation duration time (s); *C*, Time to first fixation (s); *D*, Time before the first fixation (s); *E*, Observation duration time (s); *F*, Observation count; *G*, Time to click (s); *H*, Time from first notice to click (s); *J*, Fixation count before click.

#### Stability and normality analysis of dependent variables

**Figure 6:**
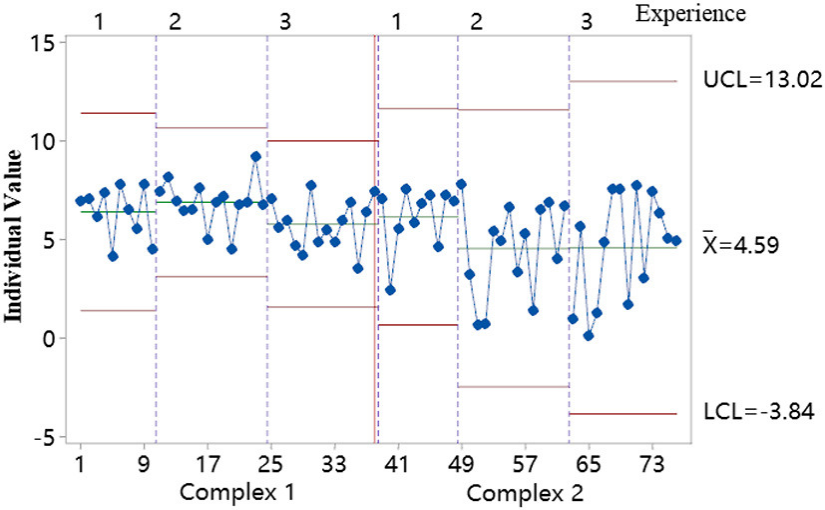
Stability chart of fixation duration.

We analyzed the data fixation duration (short for fixation duration time), performed a stability and normality test, and obtained an individual value stability chart with Minitab, as shown in Figure 6. In this chart, the reference line is 3.00 standard deviations from the center line, all the data is between the upper reference line and the lower reference line, that is to say, the experiment data fixation duration is stable.

**Figure 7:**
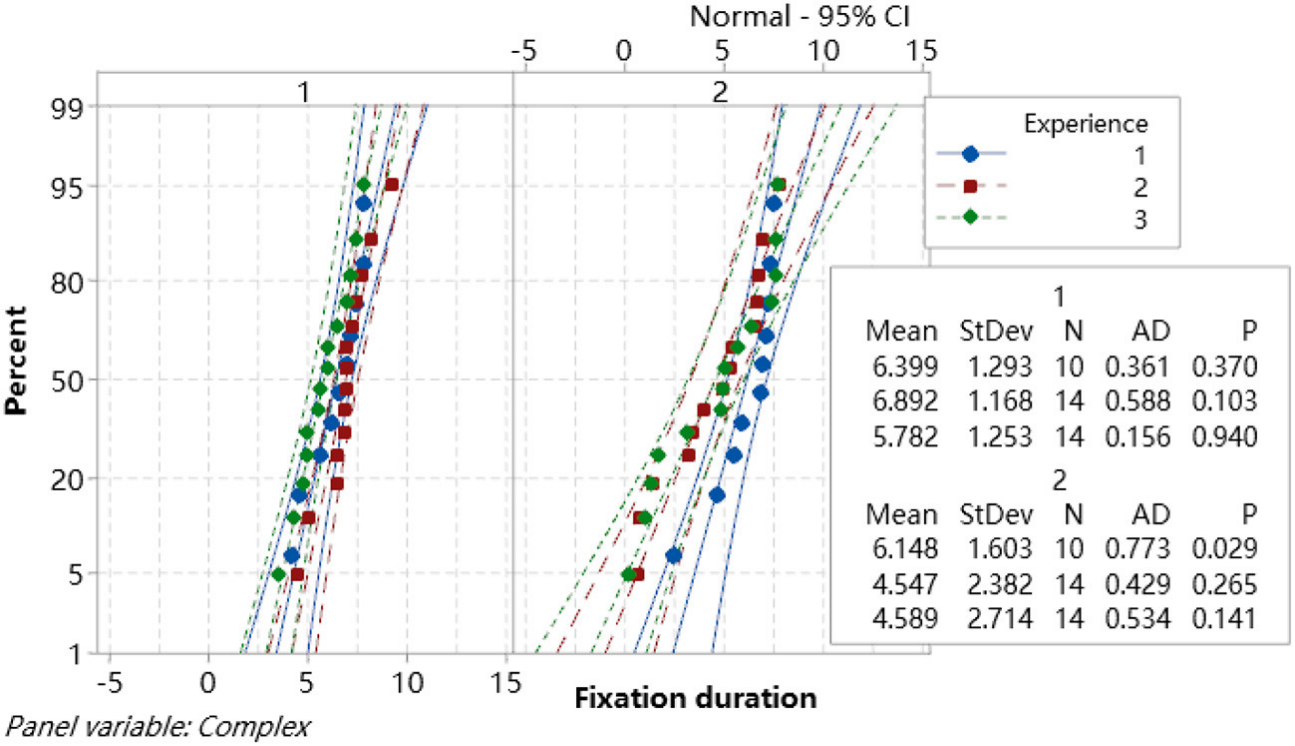
Probability plot of fixation duration.

A normality test was conducted for fixation duration data (Figure 7) and a difference test for the different group experience levels, according to the Minitab explanations. For all the data between the reference lines, *P* > 0.05 which means the data followed a normal distribution. The results showed that the experimental data fixation duration was stable and normal and met the primary demand for DOE analysis.

### Orthogonal factorial analysis

**Figure 8:**
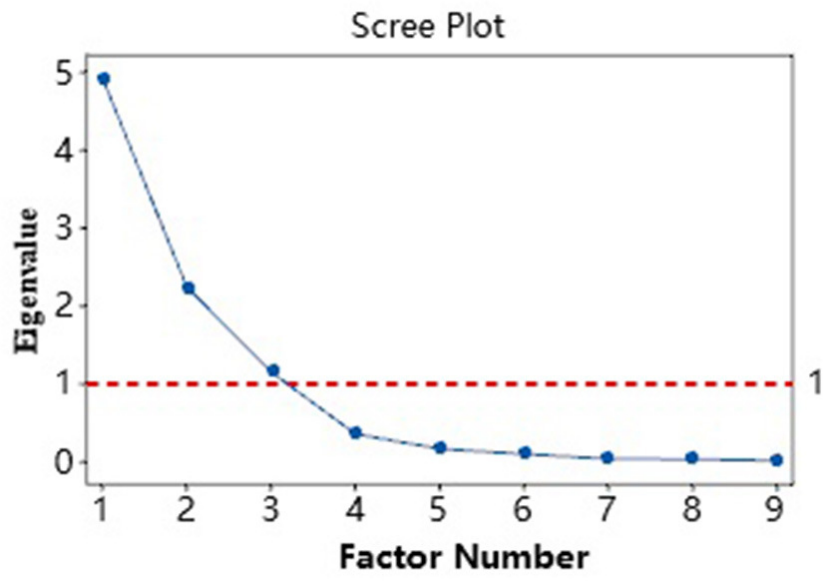
Scree plot of all the eye-tracking metrics.

The orthogonal factor analysis model helped us find the number of common factors. We performed an orthogonal factorial analysis of all eye-tracking metrics with Minitab and obtained the scree plot shown in Figure 8. The scree plot orders the eigenvalues from largest to smallest. The ideal pattern is a steep curve followed by a bend and a straight line. We found the components in a steeper curve before the first point that starts the line trend (68). In Figure 8, the Kaiser criterion's eigenvalue was more than reference line 1 which means the three common main independent factors that affected the experiment results need to be examined.

#### Experience

According to the natural working attributes of the three groups' participants, the non-obvious areas of interest (AOI) regions are Option 1 and Option 2 in test B (Figure 3). This shows that experienced personnel in the field have a wider range of vision. We tested the significance of the eye-tracking experimental data fixation duration with the three groups. Table 4 shows fixation duration vs. experience using the F-test method with 95% confidence. The two tests indicated the shortest fixation time with Group 3 participants, wherein on-site experience were the richest. The mean value of different groups with different experience levels was significantly different in test A but not in test B.

**Table 4:** One-way ANOVA: fixation duration vs. experience.

**Test**	**Experience**	**N**	**Mean**	**StDev**	**95% CI**	***P*-value**
A	1	10	6.643	1.118	(5.880, 7.406)	0.0049
	2	14	6.892	1.168	(6.248, 7.537)	
	3	14	5.782	1.253	(5.138, 6.427)	
B	1	10	5.904	1.651	(4.392, 7.415)	0.317
	2	14	4.547	2.382	(3.270, 5.824)	
	3	14	4.589	2.714	(3.312, 5.867)	

#### Chatbot safety training

Half of the participants in each group accepted our chatbot safety training. For each test, we conducted a two-sample *T*-test as the factor of the chatbot. In Table 5, both test *P*-values were more than 0.05. That is to say, our experimental stimulus was not significant in a single-factor analysis when people with different experience levels and the complexity levels of the hazards were not separately analyzed.

**Table 5:** One-way ANOVA: Fixation duration vs. a chatbot.

**Test**	**Chatbot**	***N***	**Mean**	**StDev**	**95% CI**	***P*-value**
A	N (1)	19	6.029	1.247	(5.439, 6.618)	0.123
	Y (2)	19	6.678	1.288	(6.088, 7.268)	
B	N (1)	19	5.113	2.363	(3.989, 6.238)	0.743
	Y (2)	19	4.854	2.471	(3.730, 5.979)	

#### The complexity of the test

We did a two-sample *T*-test for the fixation duration vs. the factor complex, which is tests A and B. The two test scenarios have different levels of complexity, which we express as factor complex. Table 6, shows *P* =0.003 < 0.05, showing that the complexity of the test is a significant factor for further analysis.

**Table 6:** One-way ANOVA: Fixation duration vs. complex.

**Complex**	***N***	**Mean**	**StDev**	**95% CI**	***P*-value**
1	38	6.353	1.293	(5.733, 6.974)	0.003
2	38	4.984	2.388	(4.363, 5.605)	

### Full factorial experiment result

#### Factors, levels, and pareto chart

As fixation duration is the response variable (dependent variable), we created a general full factorial experiment with three factors: experience (three levels), chatbot (two levels), and complex (two levels), as shown in Table 7. We obtained an orthogonal experimental results table using Minitab.

**Table 7:** Factors and the levels for DOE.

**Factors**	**Levels**		
Experience	Group 1	Group 2	Group 3
	1	2	3
Chatbot	N	Y	
	1	2	
Complex	Test A	Test B	
	1	2	

**Figure 9:**
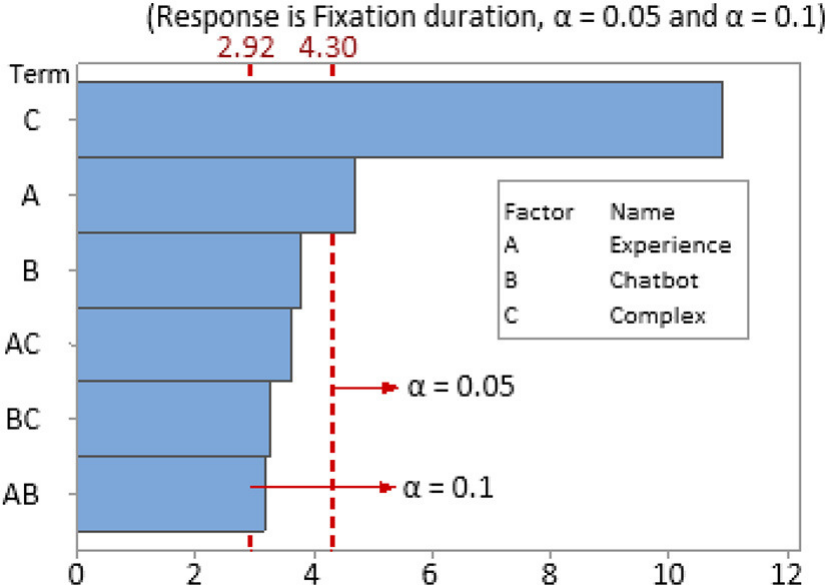
Pareto chart of the standardized effects.

After conducting factorial analysis in Minitab, we checked the optimal λ and obtained the Pareto chart (Figure 9). The confidence level for all intervals was 95% when α = 0.05 and 90% when α = 0.1, respectively. Hasanzadeh, Esmaeili (72) suggested identifying a hazard-identification experiment at a 90% confidence interval and a *p*-value < 0.1. The results were accepted as the interactive terms' *p*-values were more than 0.05 but less than 0.1. When the confidence level for all intervals was 95% (α = 0.05), the reference line value was 4.303, which means only factors A (experience) and C (complex) had a significant impact on response variables fixation duration. When the confidence level for all intervals was 90% (α = 0.1), the reference line value was 2.920. Factors A (experience), B (chatbot), C (complex), and the 2-way interaction terms AC, AB, and BC all had a significant impact on the response variable, fixation duration.

#### DOE result

The three independent variables significantly affected the dependent variables, and there was an interaction between every two variables. As shown in Table 8, the analysis of variance for the transformed response for the model was *P*-value=0.037 smaller than 0.1, with our 90% Confidence level. So, the null hypothesis was rejected, and the factorial analysis model was valid. All the linear terms P-value and all the 2-way interaction terms P-value were smaller than 0.1, meaning the model's main factors and interaction terms significantly influenced the response variable. The VIF (variance inflation factor) in this model was 1.33 and 1.0 < 10. There was no multicollinearity between factors (73). As shown in Table 9, *R*-sq =99.16% and *R*-sq (adj) = 95.40%. The two were close, so the model's regression effect was good. The R-sq(adj) value was high. This model could explain 95.4% of the response variable variation (73).

**Table 8:** Analysis of variance for transformed response.

**Source**	**DF**	**Adj SS**	**Adj MS**	**F-value**	***P*-value**
Model	9	13.8642	1.54047	26.36	0.037
Linear	4	10.4301	2.60752	44.62	0.022
Experience	2	2.6181	1.30903	22.40	0.043
Chatbot	1	0.8386	0.83863	14.35	0.063
Complex	1	6.9734	6.97341	119.33	0.008
2-Way Interactions	5	3.4341	0.68682	11.75	0.080
Chatbot*Experience	2	1.2214	0.61069	10.45	0.087
Complex*Experience	2	1.5937	0.79683	13.64	0.068
Complex*Chatbot	1	0.6191	0.61909	10.59	0.083
Error	2	0.1169	0.05844		
Total	11	13.9811			

**Table 9:** Model summary for transformed response.

***S***	**R-sq**	**R-sq (adj)**	**R-sq (pred)**
0.241739	99.16%	95.40%	69.91%

### Main effects

**Figure 10:**
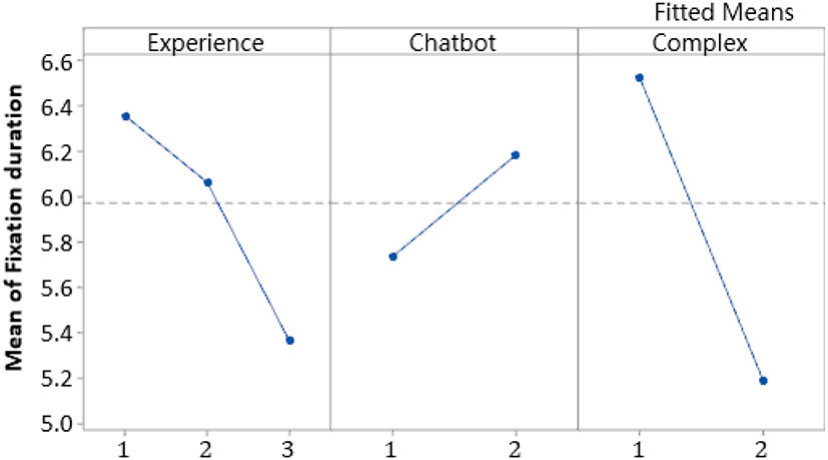
Main effects plot for fixation duration.

The main effects of the three factors are shown in Figure 10. The main effect of experience levels was *P* =0.043 < 0.1, showing that experience had a significant inverse relationship with fixation duration. In the experience level block in the main effect plot, the higher the construction site work experience, the lower the value of fixation duration, which meant less time focused on the obvious AOI. The main effect value of chatbot training was *p* =0.063 < 0.1. Participants who received chatbot training were significantly positively correlated with fixation duration, their fixation duration time was longer than those who did not. For the factor, complex, the main effect value was *p* =0.008 < 0.1, and complex was significantly inversely related to the fixation duration. The two experimental test pictures contained close and distant views. The distant-range view required more time to find the danger point and the participants allocated less time to the hazards of the AOI.

### Interaction effects

**Figure 11:**
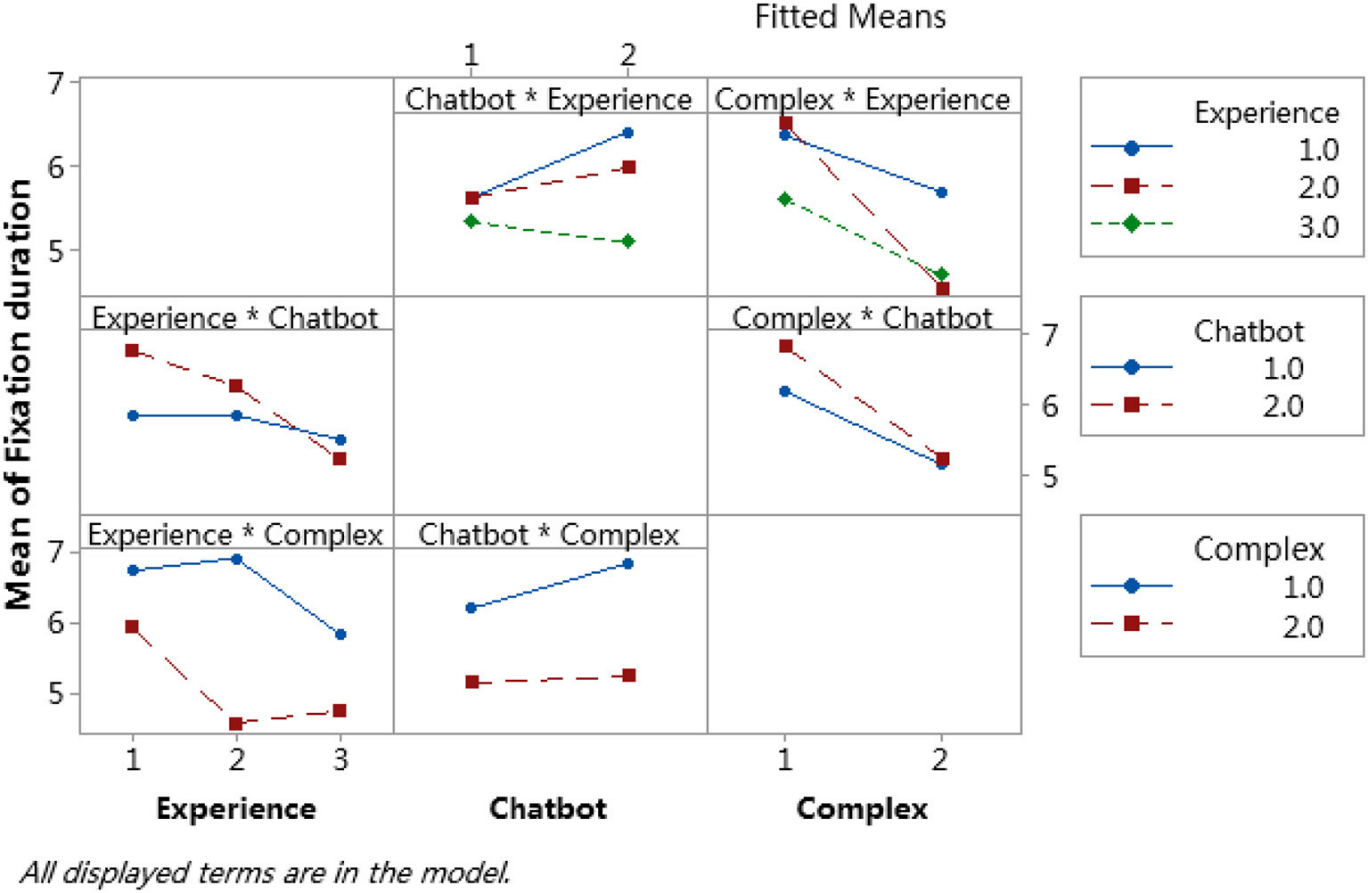
Interaction plot for fixation duration with the three factors.

**Figure 12:**
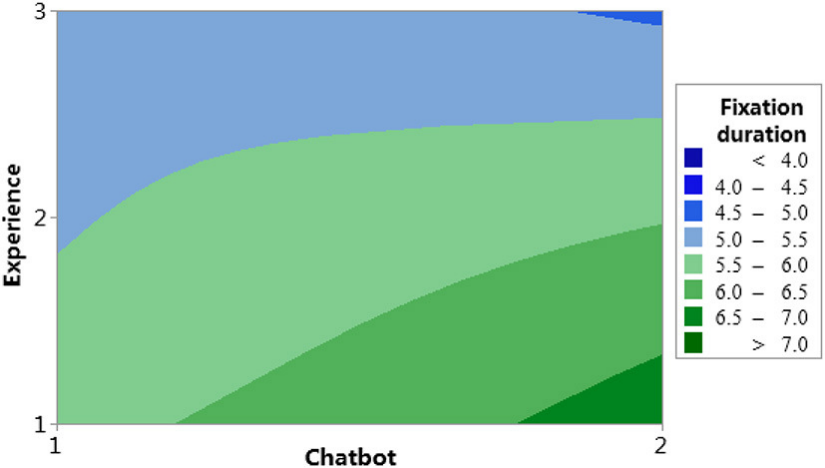
Contour plot of fixation duration vs. experience and chatbot.

**Figure 13:**
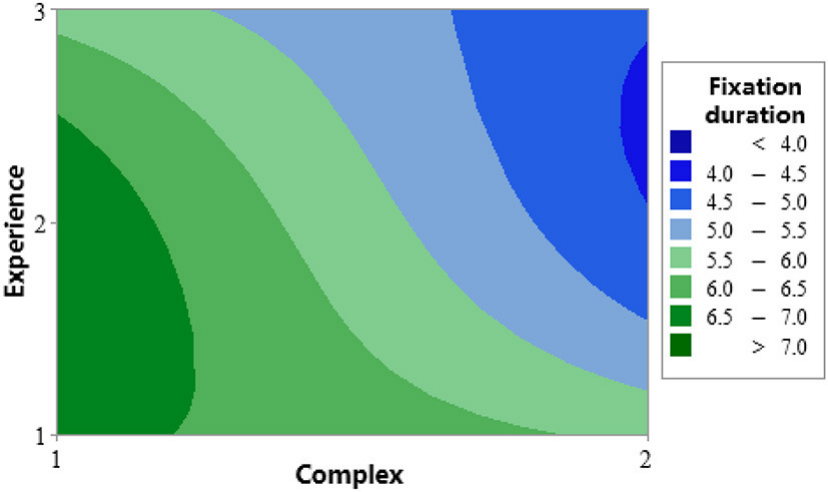
Contour plot of fixation duration vs. experience and complex.

For the analysis of the relationship of the 2-way interaction items, we focus on the upper right three blocks: Chatbot^*^Experience, Complex^*^Experience, and Complex^*^Chatbot. The interaction effect analysis for the three factors is shown in Figure 11. Figures 12, 13 are contour plots that show the visual expression of the interaction effect of the two factors on the response variable, fixation duration.

#### Chatbot and experience

In the model result for the item, Chatbot^*^Experience, the *p*-value was 0.087 < 0.1 and the interaction was significant. In Figure 11, in the Chatbot^*^Experience block, the blue line is experience level 1 (group 1), the red line is experience level 2 (group 2), and the green line is experience level 3 (group 3). All three lines value are similar when the Chatbot at the low level (Chatbot =1) and significantly different at the high level (Chatbot =2). Figure 12 shows the variation of the value of the response variable, fixation duration, with factors, chatbot and experience level. When Chatbot =2 and Experience =1, the fixation duration value is the largest, while when Chatbot =2 and Experience =3, the fixation duration value is the smallest. The same conclusion was reached that the effect of chatbot training was significantly correlated with the level of experience.

#### Complex and experience

In Table 8, the interaction item Complex^*^Experience *p*-value was 0.068 < 0.1, and was significant. In Figure 11, the Complex^*^Experience block, as complex was raised from the lower level to a high level, the fixation duration value at all experience levels significantly decreased. The novice participants' experience level 2 (red line) recorded the most significant reduction. The two lines were almost parallel when the experience was at level 1 (blue line) and level 3 (green line). In Figure 13, when complex and experience were at the lower level (Complex =1, Experience =1), the fixation duration value was more prominent with a darker green color, indicating more time on the spot in the area of the hazard. While complex and experience were both at a high level (Complex =2, Experience =2), the fixation duration value was smaller. So, the fixation duration time of the participants in the two groups showed almost the same pattern as the increase in the complexity of the test.

#### Complex and chatbot

In Table 8, the interaction item Complex^*^Chatbot *p*-value was 0.083 < 0.1, and was significant. In Figure 11, in the Complex^*^Chatbot block, when Complex at the lower level (Complex =1), the response values on Chatbot 1 (red line) and Chatbot 2 (blue line) were significantly different, meaning that the fixation duration time of the participants who had received chatbot safety training was significantly different to those who did not. Nevertheless, when the complexity level was high (Complex =2), the fixation duration value was not significantly different.

## Discussion

This research investigated the usage of Telegram chatbots for raising construction participants' hazard awareness. We experimented with three main factors, i.e., work experience, test scenario complexity, and the presence (or absence) of chatbot safety training and their interaction with the hazard awareness of construction practitioners.

### Work experience and hazard awareness

In this experiment, Group 3 had the longest site work experience, but their fixation duration times were the smallest in both eye-tracking tests. This result is close to previous studies. Dzeng, Lin (7)'s eye-tracking experiment in four workplaces found that the mean value of fixation duration of experienced workers was smaller than those of novices. Does this mean workers with rich experience pay less attention to a dangerous environment? Although Dzeng, Lin (7), combining with previous studies, Perlman, Sacks (74), Sacks, Rozenfeld (75), and Cheng and Wu (38), propose that years of construction management experience are not necessarily conducive to improving the ability of danger identification, they emphasize that experience must be directly related to construction hazard sites. We give a different explanation in that the eye-tracking metric fixation duration times with small values for a specific area indicate that the visual search range is large and workers with rich field experience have good hazard awareness. We quote a tower crane conductor at the experiment site: “We have been working at a dangerous site for a long time, so we pay more attention to the changes around us.” “We not only pay attention to the significant danger points, but also need to pay attention to the changes in the surrounding environment at any time to prevent the sudden intrusion of dangerous external factors.” Hasanzadeh, Esmaeili (50) reported that participants with more experience and a deep understanding of their surroundings had higher situational awareness too.

### Chatbot enhanced hazard awareness but was affected by other factors

Based on Ebbinghaus' forgetting curve, someone who accepted the training only had about 20% residual memory after four or five days. The main effect plot (Figure 10) showed that they had more fixation duration time on the AOI. The eye-tracking test presented a specific scenario, as in the training transfer theory studies (76) and in Namian, Albert (40)'s discussion, the trained participants unconsciously recalled the chatbot training they had and fixated their awareness on the AOI of the test image. It shows that the chatbot safety training raised participants' subconscious recognition of construction hazards and improved their hazard awareness.

The chatbot training had a more substantial impact on novices (group 2) and those who work for the construction industry but do not always need to work on-site (group 1) than on those who work on-site. That means the chatbot safety training we developed almost had no use to participants with rich on-site work experience. Dzeng, Lin (7) and de la Fuente Suárez (27)'s conclusion shows that novice and experienced practitioners have different hazard awareness, while we emphasize that the chatbots should be developed according to different work experience levels. For site novices, the simple chatbots developed in this study have a significant effect on improving hazard awareness. For different test scenarios, chatbot safety training can dramatically improve hazard awareness and recognition ability in low-complexity scenarios but has little effect on high-complexity construction scenes. In practice, the content of safety training materials could be matched with special technical details for the construction workers or practitioners so that the training can achieve good results.

### The complexity of the potential hazard

In our experiment, the novices with less experience show their hazard awareness becoming more sensitive as the complexity of the test increases, which is different from the richly experienced practitioners. Han, Yin (26) emphasized the influence of site conditions on eye-tracking results: housekeeping and proper site layout help the work subjects' cognitive load on the hazard awareness test; it is critical not only to productivity but also to safety performance. we paid more attention to workers with rich construction site experience on more complex tasks, spent less time on the spot, gave more attention to the situation, and had more confidence in the hazards of the construction site.

### Others factors

This research's experimental conditions restricted the number of factors. As mentioned in the situational awareness theory, in the research of construction hazard awareness, other factors such as cognitive load (77), mental fatigue (78), knowledge confirmation bias (25), and physical condition have been studied, so an experiment could be designed with more factors and levels. Multiple factors and levels would comprehensively represent the hazard awareness model of the construction site, and allow more empirical investigation using eye-tracking technology in construction safety studies (79) to help understand why construction hazards remain unrecognized at the work interface, and why safety training is less effective (40, 80).

Our research limitations were: the experimental design factors, eye-tracking equipment, and volunteers. This study, using AI online eye-tracking, was limited to a frequency of 30 Hz, which is not very high, meaning that this may limit the eye-tracking fixation and other data. Furthermore, perhaps with a larger number of volunteers and a higher degree of cooperation, it may be possible to obtain more useful data.

## Conclusion and future research

Given the high accident rates in the construction industry, there are many different safety training methods for improving site hazard awareness. These include traditional face-to-face training, virtual reality training, and onsite models that simulate construction sites. In comparison, chatbots are more common in the commercial sector for customer service or in language training—none of these tests the effectiveness of hazard awareness training *via* chatbots. There has been no comparison of the impact of a Telegram chatbot's effectiveness in raising construction practitioners' safety awareness between experienced and non-experienced workers. A few factors include the level of complexity of the hazard, site experience, and types of work these people engage in. Our study fills these research gaps.

Our experimental design used a low-cost chatbot as the experimental treatment factor to study the impacts of chatbot safety training on hazard awareness. On-site experience and the complexity of dangerous scenes are interaction factors. We introduced a DOE (design of the experiment) full factorial orthogonal experiment design method. The results showed that chatbot safety training could improve the novice and lesser site-experienced workers' hazard awareness even under Ebbinghaus's forgetting curve's proposition at the end of the fifth day of the experiment. Low-cost Chatbot safety training could improve site workers' hazard awareness, and the design needs to be adjusted according to participants' construction site experience and their current job. As classroom training may require many participants and costs for the trainers, and as people are used to online training after the COVID-19 pandemic (evidenced by online education providers like Coursera, EdX and online university degrees provided by universities), such results imply that a Telegram chatbot may supplement some face-to-face hazard awareness training.

## Data availability statement

Anonymized versions of the data are included within the article/Supplementary material. Further queries should be directed to the corresponding author(s).

## Ethics statement

Written informed consent was obtained from the individual(s) for the publication of any potentially identifiable images or data included in this article.

## Author contributions

XZ: conceptual idea, wrote, and reviewed and edited the manuscript. RL: conceptual idea, conceived, designed the study, and completed the paper in English. MC: conceived, designed the study, and revised the important intellectual content. KS: gave valuable research advice and revised the manuscript. All authors contributed to the article and approved the submitted version.

## Funding

The work described in this paper was fully supported by a grant from the Research Grants Council of the Hong Kong Special Administrative Region, China (Project No. UGC/FDS15/E01/18).

## Conflict of interest

The authors declare that the research was conducted in the absence of any commercial or financial relationships that could be construed as a potential conflict of interest.

## Publisher's note

All claims expressed in this article are solely those of the authors and do not necessarily represent those of their affiliated organizations, or those of the publisher, the editors and the reviewers. Any product that may be evaluated in this article, or claim that may be made by its manufacturer, is not guaranteed or endorsed by the publisher.
